# State-of-the-Art Fluorescent Probes: Duplex-Specific Nuclease-Based Strategies for Early Disease Diagnostics

**DOI:** 10.3390/bios12121172

**Published:** 2022-12-15

**Authors:** Ghazala Ashraf, Zi-Tao Zhong, Muhammad Asif, Ayesha Aziz, Tayyaba Iftikhar, Wei Chen, Yuan-Di Zhao

**Affiliations:** 1 Britton Chance Center for Biomedical Photonics at Wuhan National Laboratory for Optoelectronics-Hubei Bioinformatics & Molecular Imaging Key Laboratory, Department of Biomedical Engineering, College of Life Science and Technology, Huazhong University of Science and Technology, Wuhan 430074, China; 2 Hubei Key Laboratory of Plasma Chemistry and Advanced Materials, School of Materials Science and Engineering, Wuhan Institute of Technology, Wuhan 430205, China; 3 Key Laboratory of Material Chemistry and Service Failure, School of Chemistry and Chemical Engineering, Huazhong University of Science and Technology, Wuhan 430074, China; 4 Key Laboratory of Biomedical Photonics (HUST), Ministry of Education, Huazhong University of Science and Technology, Wuhan 430074, China

**Keywords:** duplex-specific nuclease, microRNA, single-nucleotide polymorphism, fluorescent detection, diagnosis

## Abstract

Precision healthcare aims to improve patient health by integrating prevention measures with early disease detection for prompt treatments. For the delivery of preventive healthcare, cutting-edge diagnostics that enable early disease detection must be clinically adopted. Duplex-specific nuclease (DSN) is a useful tool for bioanalysis since it can precisely digest DNA contained in duplexes. DSN is commonly used in biomedical and life science applications, including the construction of cDNA libraries, detection of microRNA, and single-nucleotide polymorphism (SNP) recognition. Herein, following the comprehensive introduction to the field, we highlight the clinical applicability, multi-analyte miRNA, and SNP clinical assays for disease diagnosis through large-cohort studies using DSN-based fluorescent methods. In fluorescent platforms, the signal is produced based on the probe (dyes, TaqMan, or molecular beacon) properties in proportion to the target concentration. We outline the reported fluorescent biosensors for SNP detection in the next section. This review aims to capture current knowledge of the overlapping miRNAs and SNPs’ detection that have been widely associated with the pathophysiology of cancer, cardiovascular, neural, and viral diseases. We further highlight the proficiency of DSN-based approaches in complex biological matrices or those constructed on novel nano-architectures. The outlooks on the progress in this field are discussed.

## 1. Introduction

Single-nucleotide polymorphisms (SNPs) are variations in base pairs at specific positions in the genome. SNPs are well-defined as loci with alleles that vary at a single base, with the infrequent allele having a rate of at least 1% in an arbitrary set of entities in a population [1]. Thus, SNPs are regarded as one of the best biological markers in case-control studies. SNPs influence a variety of physiological and pathological processes, such as cellular senescence, inflammation, apoptosis, and immune response, by altering the expression of traditional inflammation markers. Recent studies revealed that SNPs in the genes that produce microRNAs (miRNAs) regulate their expression or activity to affect different aspects of disease [2]. MiRNAs, a class of endogenous, non-coding small RNA with a length of 19–25 nucleotides, have a significant impact on the control of gene expression and have been recognized as a promising cancer biomarker candidate for early clinical diagnosis due to the connection between miRNA dysregulation and many human diseases, particularly cancer and cardiovascular diseases [3,4]. Moreover, miRNA polymorphisms have also gained more attention in recent years because they can be used as prognostic biomarkers for a variety of diseases, in addition to their involvement in the pathophysiology of diseases [5]. It is essential to create a multiplex assay for miRNA because multiple miRNAs may regulate the same gene.

Quantitative real-time PCR (qRT-PCR), microarrays, and the frequently used northern blotting are just a few of the techniques that have been developed for miRNA detection. The traditional detection method for miRNA assays is northern blotting, but it has poor sensitivity [6,7]. The short length of miRNA limits qRT-PCR and increases the difficulty of primer design. Since miRNAs have varying melting temperatures, applying microarrays to multiplex assays is challenging. Isothermal amplification techniques commonly employ polymerases and nicking enzymes to generate a large number of desired products via site-specific sequence extension and nicking. However, amplification systems are frequently more complicated than expected [8]. In addition to polymerases and nicking enzymes, sophisticated tags, templates, and nicking enzyme-specific recognition sequences are required [9]. These requirements are inconvenient for point-of-care (POC) testing and cell imaging assays, and they increase the possibility of errors and costs. This situation emphasizes the demand for simple and affordable approaches, such as biosensors [10]. The integration of biosensing devices has led to the creation of small-sized, portable, high-throughput instruments that are more sensitive, selective, and affordable [11]. These instruments are also capable of real-time and rapid detection techniques that are appropriate for POC applications. Low expression levels, small size, rapid degradation by RNase, and sequence homology always challenge the accuracy of miRNA assays [12,13].

To overcome these problems for miRNA detection, various techniques such as colorimetric, electrochemical, chemiluminescent, Raman scattering, and surface plasmon resonance (SPR) have been proposed in biosensor design [14,15,16]. Fluorescence-based techniques stand out among them because of their ease of use, high sensitivity, multiplexing, and in vivo test capacity. Quenching probes such as molecular beacons (MB) and TaqMan probes, as well as fluorescent nucleic acid stains such as SYBR Green I and YOYO, are widely applied for signal output [17]. Nonspecific binding of practically all nucleic acids to fluorescent dyes, such as ssDNA, dsDNA, and RNA, can result in substantial background signals and low specificity. On the other hand, to increase detection sensitivity, a variety of nucleic acid amplification methods have been developed, including classic PCR, loop-mediated isothermal amplification (LAMP), and rolling-circle amplification (RCA), HDA, NASBA, RPA, SDA, SPIA, etc. [18,19,20]. Though, these methods typically include intricate primer/template construction, multiple proteins, time-consuming testing processes, and background amplification that is nonspecific by nature. Therefore, the creation of simple, accurate, and speedy techniques for identifying miRNAs without the requirement for nucleic acid amplification is highly favored.

DSN is an enzyme isolated from the Kamchatka crab’s hepatopancreas that has a strong preference for cleaving a DNA portion in DNA-RNA hybrid or double-stranded (ds) DNA, and almost inactive to single-stranded (ss) DNA, or ss-ds RNA [21]. Moreover, this enzyme is capable of distinguishing between perfectly and imperfectly matched short duplexes up to one mismatch [22]. It has been widely used in molecular biology, together with full-length cDNA library standardization, deletion, recognition of genomic single-nucleotide polymorphisms (SNP), and measurable telomeric overhang recognition [23]. The DSN-based system does not require a complicated probe design or specialized equipment, in contrast to conventional methods. As a result, DSN cleavage based on DNA-RNA hybridization has been used in conjunction with different sensing technologies to detect miRNA [24], in which miRNAs are primarily detected by hybridizing a labeled DNA probe with the target miRNA. The resulting heteroduplex can then be split by DSN to generate signals [25,26], and this cleavage can occur repeatedly to achieve signal amplification while the target miRNA is still intact. MiRNA-DNA hybrids have been used to develop telomeric overhang and SNP detection assays [27,28]. Various labels such as quantum dots (QDs), fluorophores, nanoparticles (NPs), nucleotide sequences, alkaline phosphatase, DNAzyme moieties, horseradish peroxidase, etc., have been coupled in DSN-based detection [29]. It is possible to run both homogeneous and heterogeneous DSN-based assays [30,31]. The miRNA and DSN are all present in the solution in the homogeneous format. The advantages of homogeneous analysis include ease of use, minimal steric interference, and high enzymatic efficiency. In contrast, the heterogeneous assay requires fewer samples and has higher sensitivity and selectivity [32,33].

Herein, the most recent advancements in miRNA and SNP detection based on DSN-coupled signal amplification strategies are critically and thoroughly summarized, with a focus on: (i) the fundamental operating principles of these types of assays and biosensors and the unique roles and advantages of the DSN employed, (ii) advanced protocols established for high-performance miRNA and SNP detection, and (iii) future outlooks and the existing challenges of DSN-based assays and biosensors. This review aims to provide readers with a vision of the research field and a thorough understanding of the DSN-based detection techniques for disease diagnostics.

## 2. Diagnostic and Prognostic of miRNA Biomarkers

MiRNAs are crucial in the post-translational control of protein-coding genes. Any variations in their concentrations can impair cell function, which leads to diseases such as cancer, sterility, and Alzheimer’s cancers, endocrine, neurological, and cardiac diseases [34]. By affecting one or more genes, they may alter gene regulation and tissue-specific expression patterns [35]. They could therefore be thought of as specific biomarkers for a certain group of ailments, and the communication between cells is also under their control. Besides the intracellular environment, miRNAs are present (down to the femtomolar level) in biotic fluids such as plasma, tears, milk, saliva, urine, and cerebrospinal fluid [36]. These free miRNAs can be found in exosomes and microvesicles or be coupled to cholesterol molecules [37]. The expression levels of circulating miRNAs vary depending on the tissue and/or the disease. Hence, they play a crucial role in the diagnosis, evaluation of the prognosis, and monitoring of numerous diseases. Scientists have made significant progress, although quantifying miRNAs remains difficult due to their small size, high homology, low quantity, and high interference with other molecules [38,39]. Furthermore, conjugating the sample with NPs as well as incubating, combining, and sorting it with other reagents using strategies such as magnetic-activated cell sorting and fluorescence-activated cell sorting are examples of sample immobilization and preparation. In addition, to enhance separation and detection effectiveness, miRNA-capture biomolecules (oligonucleotides and proteins) can be immobilized in the capture area [40]. Enzymatic or non-enzymatic amplification is a crucial sample preparation step to increase the sensitivity of miRNA analysis. Most assays are categorized according to the type of transducer they use, which can vary in sensitivity and selectivity [41].

### 2.1. DSN Platforms for miRNA Cancer Biomarkers’ Detection

Cancer mortality rates can be decreased by early detection of cancer biomarkers. MiRNAs undergo expression changes in response to the development of various cancer types. Numerous studies have documented changes in miRNA expression in various cancers. MiRNA-191, miRNA-21, and miRNA-17-5p were found to be significantly overexpressed in all of the tumor types taken into consideration by Volinia et al. [42] in their large-scale miRNA analysis. They validated specific miRNA signatures for each tumor. The miRNAs are remarkably stable in serum and plasma samples. Therefore, circulating miRNAs were considered potential biomarker candidates in the blood. Mitchell et al. [43] discovered that serum levels of miRNA-141 distinguished between prostate tumor patients and healthy individuals. MiRNAs cannot be directly detected from cells by standard detection methods without the aid of amplification techniques. Each DNA chain can be cut out of double-stranded nucleic acids with the help of DSN. DNA chains/TaqMan probes can hybridize with target miRNAs and subsequently be cut off by DSN, releasing activated fluorophores into the solution, and this process can repeatedly occur for amplifying the fluorescence signal. Moreover, DSN amplification only needs a short reaction time and a simple procedure. Thus, it is recognized as an ideal miRNA assay [44]. Based on this basic scheme, more detection approaches have been proposed.

**Figure 1:**
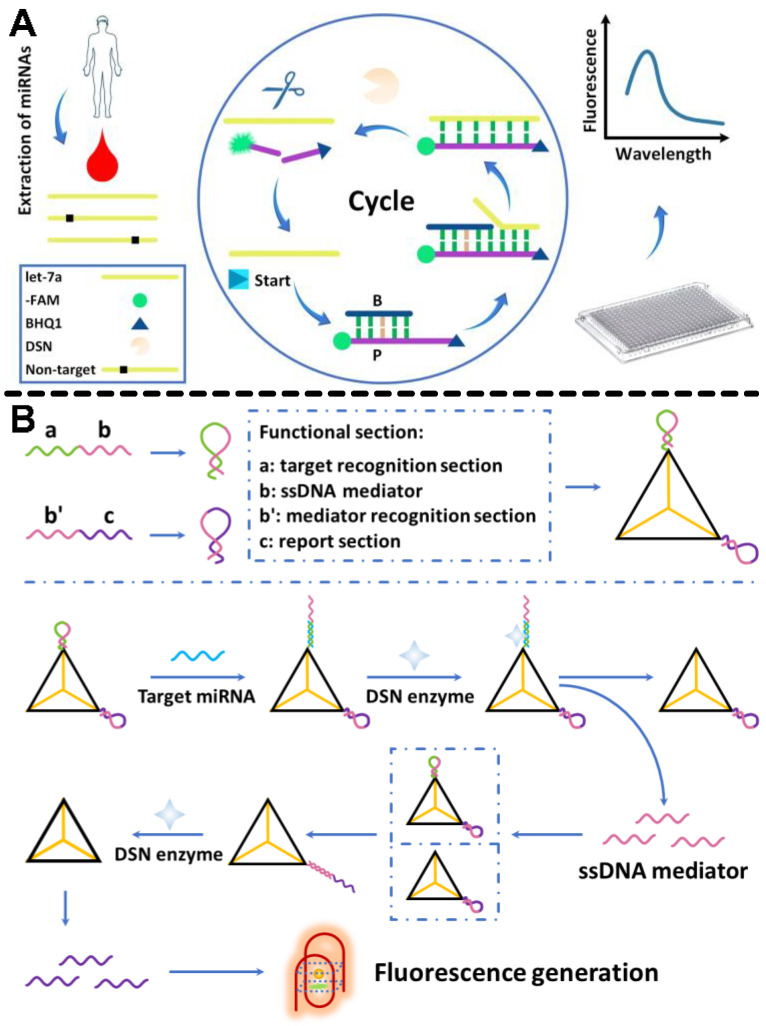
(**A**) Schematic illustration of the target invasion-triggered signal amplification for miRNA identification. (**B**) The proposed miRNA detection mechanism. Functional separation of HMBs and tetrahedral nanoprobe assembly (**top panel**). The whole operational procedure (**bottom panel**). Reproduced with permission from Refs. [46,47], respectively. Copyright 2022, Elsevier.

Recently, a dual-target recognition miRNA detection technique with enhanced specificity and equivalent sensitivity for DSN enzyme-induced signal extension was developed. High discrimination of miRNA was obtained using dual-recognition. When dual-target detection occurs, (i) miRNA forms a DNA-RNA duplex by unfolding its hairpin structure. The DNA section was digested by the DSN enzyme that released miRNA to take part in the subsequent recycling. (ii) After the DNAzyme-based nicking site formed on the loop section of the MB, miRNA attached to it, causing the MB probe to slowly unfold and produce fluorescence signals. The ability to distinguish between homogeneous miRNAs with even a single base pair mismatch was obtained using this general principle, opening the door for promising advancements in the clinical diagnosis and treatment of critical pancreatitis [45]. It was recently reported that a fluorescent sensor with cyclic signal amplification and toehold-mediated target invasion was used. They combined the toehold-mediated target invasion mechanism with the particular cleavage features of DSN to greatly boost the assay sensitivity. Throughout the experiment, the limit of detection (LOD) for the target let-7a is as low as 9.00 fM, with a decent linear range of 1 pM–100 nM and a 60 min analysis time (Figure 1A). The technique realized single-base identification in the miRNA let-7 families and was reasonably selective in detecting mismatched miRNAs. The method was utilized to detect miRNAs isolated from human serum. The results were in line with those obtained using the quantitative reverse transcription-polymerase chain reaction (qRT-PCR) method, which has numerous potential uses in biological research and clinical molecular diagnostics [46].

Similarly, a novel method for label-free miRNA detection using a DNA tetrahedral structure as a functional starting point for subsequent signal amplification using DSN enzymes was reported. When the target miRNA was present, two hairpin structure probes on the two terminals of a tetrahedral structure nanoprobe began a DSN enzyme-based recycling process. This method was based on G-quadruplex, which showed great promise for target detection without the need for labeling or expensive equipment. Highpoints of the methods include (Figure 1B): (i) a promising detection sensitivity employing DSN-based signal amplification, (ii) label-free fluorescence signal generation based on the G-quadruplex complex, and (iii) a constant signal production even under challenging experimental conditions based on the tetrahedral structure. Ultimately, the assay provided great potential for sensitive and specific miRNA quantification in clinical diagnosis and biomedical research [47].

To explore the phenomena that occur in the local microenvironment around the MB-tethered DNA probe, a highly sensitive and selective fluorescence sensor using microRNA-21 detection was developed. The effects of various DNA spacers, base-pair alignments, and surface concentrations on DSN-mediated target recycling using the aforementioned method were investigated. Under ideal conditions, a miRNA-21 detection limit as low as 170 aM was recorded. Additionally, this technique exhibited excellent selectivity in a fully matched target as compared to a single-base mismatch, and miRNAs in serum with improved recovery were realized. These results were explained by a synergistic interaction between the neutral DNA spacer triethylene glycol and the DSN enzyme, which increased the sensitivity of miRNA sensing [48]. Finally, this method eliminated the enzyme-mediated cascade reaction employed in prior studies, which is more expensive, time-consuming, and less sensitive.

Combining the powerful discriminating capabilities of MB and DSN, MB-based signal amplification may discriminate between homologous miRNAs with just a single nucleotide change [49]. Utilizing MB in a DSN system is challenging since the DSN can cleave the dsDNA portion in the MB, resulting in false-positive signals. However, complex and expensive techniques are reported, such as creating a stem for a G-quadruplex structure rather than a duplex stem or replacing the backbone of the MB stem with 2-OMe-RNA [50]. The labeling of MB ends with particular dyes, further increasing the complexity and cost of the assay. An up-front and cost-effective label-free MB based on G-quadruplex (MBG4) was created and used in the DSN system by Tian et al. [51]. In this instance, the stem was locked by the G-rich region and was unlocked by the target miRNA. A measurable signal was created when the released G-rich sequence interacted with a small-molecule ligand to form the G-quadruplex. The long stem of MBG4 must have more than 8 base pairs to avoid the development of the G-quadruplex, which is longer than the previously established minimum recognition length of DSN (no more than 6 base pairs). The significant sequence homology among miRNA members complicates miRNA analysis. An innovative method for the detection of miRNA was developed based on the MBG3 and DSN signal extension. This particular MBG3 had a special G-triplex probe. Due to the excellent controllability of the G-triplex probe, which prevented DSN digestion, the MBG3 was able to have a short stem. Nevertheless, the majority of DSN signal amplification methods have successfully minimized false-positive signals brought on by DSN cleavage without the need for additional adjustments. Owing to the enhanced identification of capabilities and DSN’s sensitive substrate selectivity, this novel technique proved suitable for high-selectivity miRNA detection. The signal response was lowered from 37% to 8% when comparing the standard linear ssDNA probe-DSN method to similar miRNA sequences with a single base modification [52].

Nevertheless, DSN amplification on paper devices (µPADs) is much more challenging to implement than in containers. The large unspecific surface area and paper fiber’s strong capability to absorb nucleic acids may largely increase the detection limit of miRNAs for analysis. Additionally, compared to the assay that occurred in solutions, the regent storage and fluorescence signal collection on paper differ. Designing PADs and creating a scheme for DSN amplification on PADs are therefore crucial for a quick, inexpensive, and accurate assay of miRNAs. For this purpose, microfluidic µPADs based on laser-induced fluorescence were developed for miRNA detection. An interface was created and used to obtain a stable fluorescence signal. To improve the sensitivity for miRNA-31 and miRNA-21, DSN amplification was performed on a double-layer µPAD. The complete analysis including sample heating was finished in 40 min. Only 1–1.5 zmol of miRNA were consumed with detection limits for miRNA-31 and miRNA-21 of 0.5 and 0.2 fM, correspondingly. When tested against mismatched miRNAs, the method showed good specificity. Finally, the technique was used to find miRNA-21 and miRNA-31 in HeLa cells, lysates of A549 cancer cells, as well as hepatocyte LO2 [53]. Breast cancer is one of the most prevalent malignant tumors in women, and miR-21, a key breast cancer biomarker, can aid in early identification. Recently, a 2D nanomaterial molybdenum disulfide (MoS_2_) was employed to create an efficient, sensitive, and intensive fluorescence sensor for the detection of miR-21. The fluorescence assay was created by combining a fluorescent dye-labeled DNA probe with MoS_2_. The sensor was then individually exposed to non-complementary miRNA, one-base mismatched miRNA, and complementary miR-21 so they could hybridize with the DNA probe. MiR-21 was detected by contrasting the fluorescence signal before and after DNA-miRNA hybridization. Complementary miR-21 was successfully distinguished from non-complementary miRNA and one-base mismatched miRNA by the assay. This assay was capable of detecting miR-21 down to a concentration of 500 pM and could complete the task in around 40 min.

### 2.2. DSN Platforms for miRNA Cardiac Biomarkers’ Detection

Cardiovascular disease is the leading cause of sickness and death worldwide. MiRNAs play crucial roles in the development of the cardiovascular system, even though genetic mutation and cellular mechanisms have long been known to contribute to the pathogenesis of a range of cardiovascular disorders [54]. MiRNA dysregulation has been associated with cardiac diseases such as heart failure, congenital heart disease, myocardial ischemia, hypertension, and atherosclerosis [55]. Due to their cell or tissue specificity, resistance to deteriorating elements or blood enzymes, and fast-release kinetics, miRNAs have the potential to be substitute markers for the initial and accurate diagnosis of disease. The regulation of cardiovascular system development, cardiogenesis, cardiac cell proliferation and differentiation, cell growth and integrity, and cardiac cell communication is regulated by miR-195, miR-126, miR-590, etc. [56]. The DSN-mediated signal augmentation technology effectively eliminates the possibility of nonspecific amplification with a simple reaction scheme as compared to traditional nucleic acid intensification methods for microRNA testing.

Ma et al. [57] developed a fluorescent method for the sensitive detection of microRNA let-7a based on pyrene excimer shift and DSN-assisted target recycling. A probe-microRNA heteroduplex formed upon hybridization with the probe when the target let-7a was present. Target let-7a was released when DSN fragmented the probe component in the heteroduplex. Let-7a was then released and hybridized with another probe, leading to the cyclic digestion of several probe strands. The reporters remained in the on-state owing to probe digestion by DSN. Pyrene excimer on-state was easily tracked using fluorescence measurements (emission at 485 nm).

To detect miRNAs, researchers have recently developed several protocols that combine the DSN enzyme and magnetic beads. However, the interfacial phenomenon underlying surface-based hybridization and target recycling with DSN assistance is still not well-understood. A potentially fatal condition, myocardial infarction, frequently results in heart failure. It is critical to establish therapeutic goals at an early stage if heart failure is to be avoided. In human embryonic stem cells, overexpression of members of the miRNA let-7 family promotes maturation caused by a metabolic switch. The smartphone’s image processing technology makes the POCT analysis procedure more sophisticated and useful since it can recognize different types of coded images while simultaneously decoding the images with great precision. As a result, it is possible to detect numerous targets while also speeding up and improving the detection accuracy of the study [58].

Meanwhile, hydrogel microparticles with varied coding modes were fabricated using flow lithography for the aim of recognizing specific miRNAs. Sandwich immunoassays revealed that different hydrogel structures had different fluorescence intensities depending on their targets. Firstly, the miRNA was captured using a hydrogel and nucleic acid mixture. To identify various miRNAs, photocuring technology was employed to create various encoded hydrogel microparticles. To create various coding forms, distinct shapes of hydrogel microparticles were produced by shining ultraviolet light through variously shaped masks. In addition, hydrogel had less nonspecific binding, which helped identify targets in complicated biological samples. The complementary pairing was used to combine miRNA with nucleic acid aptamers.

**Figure 2:**
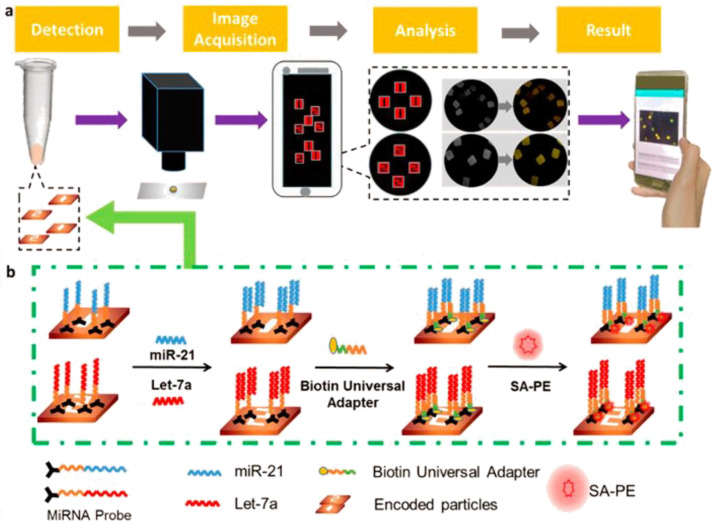
(**a**) Schematic illustration of how miRNA (miR-21, let-7a) can be detected utilizing hydrogel microparticles that have been encoded with certain shapes using a smartphone detection platform. To learn more about the kind and concentration of the miRNA, the liquid system was moved to a solid substrate after the sandwich structure formation. (**b**) Images were then taken using a self-made POCT device and analyzed using Android software. Reprinted with permission from Ref. [59]. Copyright 2019, American Chemical Society.

**Figure 3:**
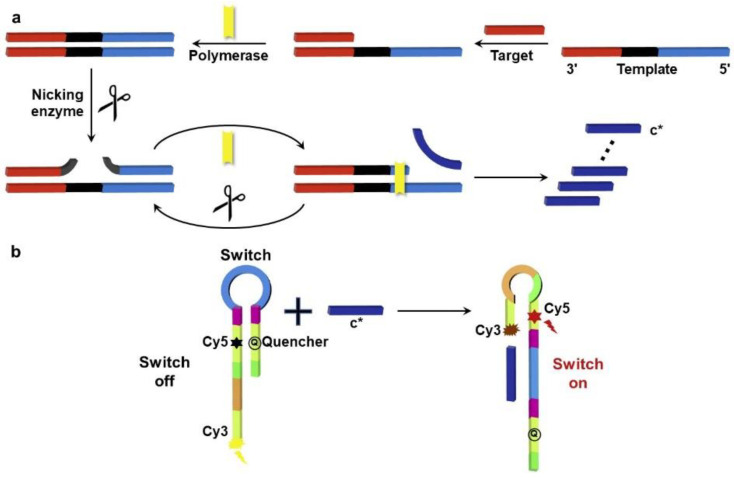
(**a**) Switch-conversional ratiometric fluorescence biosensor for miRNA let-7a detection, (**b**) illustration of exponential amplification reaction (EXPAR) and switch conversion. Reproduced with permission from Ref. [60]. Copyright 2020, Elsevier.

Phycoerythrin was also employed as a detecting signal at the same time. Using a self-built POCT device and a self-written app, images of the coding and detecting signal were obtained. Finally, the smartphone displayed the type and level of miRNA (Figure 2) [59]. However, the results are less reliable since they are prone to false-positive or false-negative detection results because majority of these fluorescence assays depend on a single signal shift. Thus, the ratiometric fluorescence detection technique has gained immense importance. Ratiometric fluorescence can be effectively carried out using Förster resonance energy transfer (FRET). By using amplicon fragments to modify the structure of the switch, the researchers created a novel switch-conversational ratiometric fluorescence assay for sensitive miRNA let-7a detection. The DNA switch was modified with a complexly designed single-strand DNA with a stem-loop construction, both of which ends were modified with fluorophores (Cy3 or Cy5) and one quencher at definite switch positions. As shown in Figure 3, an exponential amplification reaction yielded amplicon fragments (c*). When the c* hybridized with a DNA switch’s loop, the switch’s structure changed, and FRET occurred between Cy5 and Cy3. Then, two separate fluorescence signals would be generated. This innovative design has a wide range of potential applications since it can quantitatively and quickly detect the target miRNA with a low detection limit of 70.9 fM by utilizing the ratio of the two signals [60]. These strategies are versatile, making them useful for various DNA and RNA determination tasks.

Ye et al. [21] developed a sophisticated one-step DSN signal intensification technique based on a linear TaqMan probe that allows for the sensitive detection of 100 fM miRNA-141 in about 30 min. The test specificity was further enhanced by the use of the hairpin-structural probe. MiRNAs might be sensitively identified by fluorometry in batches or even in fixed cells by combining nanomaterials such as tungsten disulfide nanosheets, Au NPs, and graphene oxide (GO) with DSN-aided target recycling [61,62].

The main mechanism in these complex methods is the DSN-mediated response, which holds two intrinsic limitations. (1) Reactions on linear DNA probes demonstrate little selectivity even when there are four base differences between homologous oligonucleotides. Even though the hairpin probe can improve DSN specificity, it typically requires dedicated modification, such as via 2-OMe-RNA, which is costly and time-consuming. The delayed hybridization kinetics will also dramatically reduce the amplification efficiency. (2) To increase the amplification efficiency, the DSN system is constantly heated to 60 °C, which is higher than the ambient or body temperature. It recommends that testing using the DSN method can be conducted at a constant temperature without the use of a thermal cycler, though a heating apparatus is still necessary to maintain the required temperature for effective amplification. Due to such high temperatures, studies involving living things are challenging. In this regard, a short-probe-based DSN approach was reported, in which the hybridization rate between the target miRNA and the DNA probe had a substantial effect on the dynamics of the DSN method in light of the rate-determining phase of the DSN process via different types of probes. By carefully examining the intensification reaction on numerous DNA probes, it was found that the dynamic forces of the amplification process are greatly impacted by the annealing rate between the probe and the target miRNA. By simply shortening the DNA probe, the ground-breaking DSN approach with greatly increased specificity at 37 °C without compromising the amplification efficiency was achieved. MiRNA let-7a was sensitively identified with a limit of detection as low as 30 pM and a specificity that was 10^2^–10^4^ times higher than that of typical DSN-based approaches. The rt-qPCR approach showed good agreement with the let-7a analysis in the lysates of A549 and BEAS-2B cells. Additionally, the endogenous let-7a in A549 and BEAS-2B live was clearly observed without affecting the natural morphology of cells [63]. This technique offered a basic concept for expanding DSN-related signal amplification techniques for use in living cells and POCTs, and it will have a significant influence on the creation of quick and easy molecular diagnostic applications for short oligonucleotides.

### 2.3. DSN Strategies for miRNA Neural Biomarker Detection

Dramatic brain abnormalities and brain malignancies are caused by defects in the genetic processes that control key components of normal brain development, such as predecessor cell proliferation, cell differentiation, and apoptosis. Brain tumors are started and maintained by a specific subpopulation of stem cells, or tumor-initiating cells, which share many characteristics with healthy progenitor cells, re-enter cell growth, and can also give rise to segregated daughter cells. A wide range of regulatory systems regulates the proliferative behavior, lineage, and fate of brain progenitor cells in a precise spatial-temporal manner.

MiRNAs are an important type of molecular regulator. As we realize that brain tumors share molecular and cellular properties with healthy brain development, including the regulation of miRNA, new paths for studying and treating these brain diseases are being discovered [64]. The brain expresses a significantly higher concentration of miRNAs than other tissues. Neurodegeneration may result from the disruption of synaptic networks through structural and functional breakdown. In neurodegenerative illnesses, brain function steadily deteriorates, leading to a variety of syndromes with overlapping traits that are greatly impacted by genetic and environmental variables. For example, some disorders, such as Alzheimer’s disease (AD), multiple sclerosis, frontotemporal dementia, vascular dementia, and dementia with Lewy bodies, are associated with cognitive abnormalities. In a similar vein, the motor system is affected by Huntington’s disease, Parkinson’s disease, and spinocerebellar ataxias [65,66]. miRNA expression is upregulated in the early stages of neurodegeneration, while miRNA expression steadily declines during oncogenesis. Peripheral blood from Alzheimer’s patients has been found to have uncontrolled tiny non-coding miRNA sequences. Some other common miRNAs such as miRNA-9, miRNA let-7, miRNA-128, miRNA-124, miRNA-29, and miRNA-34abc have been linked to both brain cancers and neurodegenerative ailments [67,68,69].

DSN was used to increase the sensitivity for detecting miRNAs and reduce the amount of material consumed by increasing the target to probe hybridization numbers [70]. Liu et al. [71] combined the target specificity of chimeric MB with DSN signal amplification capability for glioma biomarker miRNA-204 detection. They designed MB with an RNA stem that comprised a loop complementary to the target sequence. Under an optimized reaction environment, this method presented the capacity to discriminate miRNA-204 sequences comprising mismatched bases. This could be an auspicious tool for specific in vitro miRNA analysis. They also employed qRT-PCR to identify the miR-204 levels in a subset of 12 samples to validate the tissue test results. The results were found to be compatible with the trend of MB-DSN detection by examining the relative expression levels estimated from the *C_t_* values of the amplification profiles of miR-204 and RNU6B. These consistent outcomes from the two distinct approaches showed that the two-step approach can quickly and precisely detect the quantities of microRNA in tumor samples.

**Figure 4:**
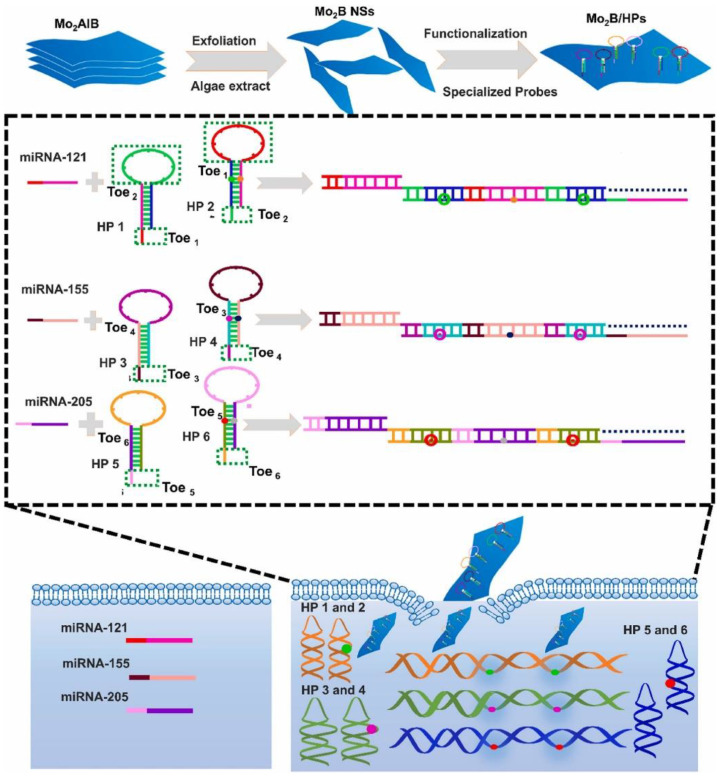
Schematic representation of a fluorescence-quenching platform based on Mo_2_B nanosheets for imaging multiple miRNAs in living cells utilizing HCR amplification. Reprinted with permission from Ref. [72]. Copyright 2022, Elsevier.

**Figure 5:**
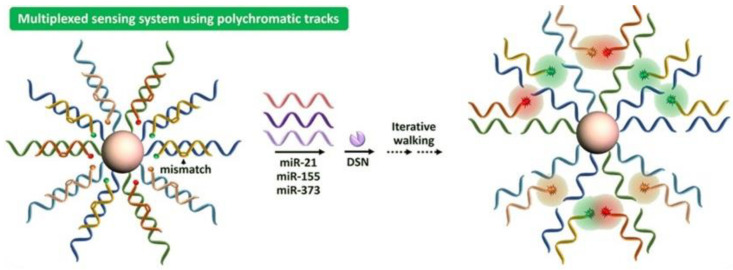
Schematic representation of the multiplexed sensing system using polychromatic tracks. Reprinted with permission from Ref. [73]. Copyright 2021, Elsevier.

It has been demonstrated that intracellular miRNA imaging is essential for elucidating both their healthy and pathological roles. Finding sequence-specific miRNAs in living cells remains a key issue. To overcome this challenge, Mo_2_B NSs, fluorescence quenching, and hybridization chain reaction (HCR) were used to develop a straightforward platform for imaging multiple intracellular miRNAs. The Mo_2_B nanosheets offered strong interactions with the hairpin probes, the ssDNA loop, and several fluorescent dyes. Following transfection, the hairpin probes recognized certain miRNA targets, activating the corresponding HCR to produce sizable DNA-miRNA duplex helix structures that disengaged from the Mo_2_B nanosheet surface and provided intense fluorescence for miRNA identification (Figure 4) [72]. The great sensitivity allowed for the imaging of multiple miRNA biomarkers in different cell types to distinguish between cancer and healthy cells. MiRNAs’ regulated expression changes in cancer cells were successfully monitored. The potential of DNA molecular machines for biosensing, drug delivery, and cellular imaging has generated a great deal of interest. Therefore, a DSN-driven nanowalker with a 13 nm-diameter AuNP was functionalized with densely mismatched DNA duplexes so that it may move gradually and autonomously along a spherical three-dimensional track. The motion is initiated by an RNA walking strand and is suppressed in the absence of the DSN because it is inactive toward the mismatched DNA duplexes. A perfectly matched DNA-RNA hybrid is created when the walking strand is added, causing a displacement response between the mismatched duplex and the walking strand. The walking strand is then released from the DNA/RNA hybrid, and DSN cleaves it at the same time, enabling it to move independently along the track. For the extremely effective operation of 3D nanowalkers, this study provided a revolutionary energy input and power approach. The proposed nanowalker also exhibited femtomole-level sensitivity in single and multiplexed sensing of three miRNA targets and may be constructed in a target miRNA-specific manner by altering the mismatched duplexes. Furthermore, multiplexed quantification of the three miRNAs in biological samples was accomplished (Figure 5) [73], further indicating the enormous potential of the proposed nanowalker in biomedical research and early clinical condition.

The hsa-miR-205, which is also known as miRNA-205 and contains 22 bases, lowers drug resistance and raises cells’ receptivity to pharmacological treatments. Additionally, miR-205 is used in both biomedicine and nano-therapy and is essential for chemosensitization. Since radiation-resistant tumor cells have substantially higher levels of miR-205 than normal cells, recent investigations have demonstrated that miR-205 is associated with radioactivity in nasal and brain carcinomas. It is essential to be able to detect miR-205 with great sensitivity because this discovery could aid in the individualization of NPC treatment. In this case, a method for miR-205 detection was developed using the graphene oxide sensor and DSN for fluorescence signal amplification. The researchers used a target-recycling method in which the action of a single miR-205 target in the presence of DSN resulted in the cleavage of multiple DNA signal probes. The technique demonstrated its ability to analyze miR-205 in solution by having a linear range of 5–40 nM and the ability to identify miR-205 at concentrations as low as 132 pM. The approach was so exact that it could distinguish between a target miRNA and sequences with single-, double-, and triple-base mismatches, as well as between distinct miRNAs. Due to its ease of use and high sensitivity/specificity, it has the potential to be used in radioresistance research and early clinical cancer diagnosis [74]. 

### 2.4. DSN-Based Additional Strategies for miRNA Detection

Nanotechnology advancements have opened up new possibilities for developing the next generation of nucleic acid sensors. In fact, by combining DNA nanotechnology, advancements in nuclease-based signal amplification, and functional NPs, new assays with advantageous properties can be developed. A novel miRNA assay using DSN, simple DNA probes, and fluorescent QDs was recently reported [75]. In an isothermal target-recycling approach, multiple DNA signal probes were cleaved in response to a single miRNA target. The incorporation of DNA-functionalized QDs allowed quantitative fluorescent readout by FRET-based interaction with the DNA signal probes. Target recycling resulted in a 3-fold increase in amplification, which increased the miR-148 detection limit to 42 fM with improved selectivity versus other miRNAs and mismatched sequences. Similarly, a method for miRNA detection was developed that combined the DSN, Exo-III enzyme-assisted signal extension, and DNA-templated Ag NCs for signal production. The method ultimately demonstrated a broad detection range, across 5 orders of magnitude. The high signal emission capacity of Ag NCs and dual-signal recycling helped to obtain the detection limit of 245 aM. The method’s excellent selectivity for miRNA-21 detection holds potential for use in biosensing and medical analysis [76]. Novel sensors have great potential for quickly and accurately determining miRNA. However, more efforts should be made to improve the anti-interference aptitude and analytical performance in biological matrices. Miao et al. [77] reported a fluorescent sensor based on a DNA structural switch and DSN-mediated magnification. Furthermore, AuNPs were employed as a 3D reaction interface and fluorescence quenchers. The developed sensor displayed high selectivity and a low detection limit (0.1 fM) for miRNA let-7a. The developed detection approach offered a powerful instrument for medical study and could be employed for cancer analysis.

However, using a dual-mode sensor instead of a single analysis has various benefits. It focuses on combining two different sensors and performing subsequent data analysis using appropriate statistical models to significantly increase the resolving power, precision, and replicability of analytical quantities. It has taken a lot of work to create innovative, promising sensors with dual-mode output for miRNA detection. Yu et al. [78] reported a flexible dual-model biosensor for miRNA detection based on the quenching effect of graphitic carbon nitride towards Pd NCs. This biosensor employed the DSN-assisted signal extension method. Only a few attempts have been made to quantify tumor-related miRNAs via dual-mode biosensing and DSN-assisted target recovering amplification approaches. As a result, a fluorescent and colorimetric dual-sensor for sensitive miRNA detection was reported [79]. This system enabled the one-base discrimination capability of DSN that exhibited high selectivity. To ensure high sensitivity and low background, the employed hairpin probes were intricately designed with anti-digestion properties under optimal conditions. The fluorescence intensity displayed a linear relationship against the miRNA-21 with a 50 pM detection limit. The absorption intensity for colorimetric analysis exhibited a 3 fM detection limit for miRNA-21 concentration. Furthermore, miRNA was successfully detected in a range of cell lysates employing the dual-mode sensor, generating new opportunities for the quantification of biomarkers in biomedical and medical diagnostics.

Mass spectrometry (MS) is a particularly useful tool because it can reveal the molecular weight. It can differentiate a difference of a few Daltons in molecular weight with exclusive isotope patterns, and MS has the potential to offer the most definite statistics and be employed in complex assays without necessitating analyte labeling [80,81]. Recently, a method for quantifying miRNA was developed using LC-electrospray ionization tandem MS (LC-ESI-MS/MS). The multistage signal intensification method comprised reporter molecule acid hydrolysis to yield unrestricted nucleobases, target recovering augmentation with a DSN, and miRNA target improvement with a DNA probe-magnetic bead couple. LC-ESI-MS/MS was employed to precisely and constantly record the subsequent nucleobases. Using miRNA-21, the current protocol was applied to study biological samples together with cell cultures and serum. The findings confirmed that miRNA targets could be easily and affordably quantified using the recognized LC-ESI-MS/MS system without total RNA isolation from the sample. The detection limit for the miRNA-21 was 60 fM, and the content of miRNA-21 was reduced to 50% in MCF-7 cells conserved with toremifene, a prevailing inhibitor of breast cancer, and the reduction was dose-dependent [82]. 

Owing to the exceptionally high sensitivity, SERS has recently emerged as one of the potential methods for miRNA analysis. Due to their high spatial and spectral resolution, benchtop confocal Raman spectrometers are frequently used for the SERS analysis of living samples in a liquid medium [83]. The most popular NMs for SERS-active substrates are AuNPs. The use of sophisticated nucleic acid intensification techniques is made easier by a pure substrate. Yang et al. [84] developed a miRNA-155 detection platform by employing a DNA microcapsule that responds to miRNA and Si@Nafion@Ag SERS-active substrate. With the help of DSN, Raman dye was released from the TB@CaCO3 composite, and since there are numerous AgNPs on the Si@Nafion@Ag substrate, a strong Raman signal was obtained. Similarly, Pang et al. [85] reported a DSN-mediated SERS biosensor for miRNA-10b detection. They measured the levels of miRNA-10b in various samples, and the results revealed that microRNA-10b levels in exosomes from patients with pancreatic ductal glandular cancer were about 59-fold higher than healthy individuals and 6-fold higher than from patients with enduring pancreatitis, while miRNA-10b levels in plasma samples from pancreatic ductal glandular cancer patient samples were about 63-fold higher. Plasmonic detection can improve end-point signal exposure owing to the hotspot development located on NSs substrates. By merging NMs collecting approaches with diversity of nucleic acid intensification methods, low levels of miRNAs in biotic fluids can be delicately detected [86]. In turn, simple, efficient, and robust practices that satisfy the distribution of POC testing, particularly in low-resource locations, are still immediately desirable in the study of clinical samples. By resolving existing optical sensing challenges and collaborating with clinicians and biochemists, plasmon-boosted biosensors have an auspicious future in therapeutic diagnosis and will open avenues for miRNA detection and appropriate clinical treatment. The different DSN-based signal amplification methods are shown in Table 1.

**Table 1:** DSN-based methods for miRNA detection.

Method	Target Analyte	Detection Limit	Real Sample	Reference
Fluorescence	miRNA-21 and miRNA-31	0.17 and 0.062 fM	CA549 and HeLa cells	[53]
Fluorescence	miRNA-21	258 pM	MCF-7 cells	[87]
Fluorescence	Let-7a	0.26 pM	A549 cells	[88]
Fluorescence	miRNA-3188	25 fM	CNE-1 cells	[89]
Voltage-assisted liquid desorption electrospray ionization tandem mass spectrometry	miRNA-21	0.25 pM	Mouse peritoneal macrophage	[90]
SERS	miRNA-21	42 aM	A549, MCF-7, HeLa, and HepG cells	[91]
SERS	miRNA-21	0.1 fM	A549, 293T and HeLa cells	[92]
Electrochemiluminescent/electrochemical	miRNA-499	28.75 aM	Human serum	[93]
Electrochemical	miRNA-21	4.5 fM	HeLa, MCF-7 cells	[94]
Electrochemical	miRNA-21	0.28 fM	Human serum	[95]
Colorimetric	miRNA-21	29 fM	Human serum	[96]
Colorimetric	miRNA-122	7.7 fM	NA	[97]

## 3. Diagnostic and Prognostic of SNP-Related Biomarkers

The variations in the sequence at specific locations in the genome are known as single-nucleotide polymorphisms (SNPs). A genetic variation at a single-base pair (bp) locus is not an SNP unless at least two alleles are present in a substantial population of unrelated individuals and have frequencies of more than 1%. The human genome is haploid and has 3 billion bp. Estimates place the number of SNPs at between 10 and 11 million, or 1 SNP for every 275 bp. Majority of SNPs only have two alleles due to the extremely low mutation rate at specific bp positions and the rarity of two-point mutations occurring at the same site over time [98].

SNP-related biomarkers are the best option for developing a large collection of polymorphic biomarkers that may be used to investigate the association between the biomarkers and a particular characteristic or disease. SNPs are useful in forensic genetic investigations for several things, including determining ethnicity, traits of people, or diseases [99].

**Figure 6:**
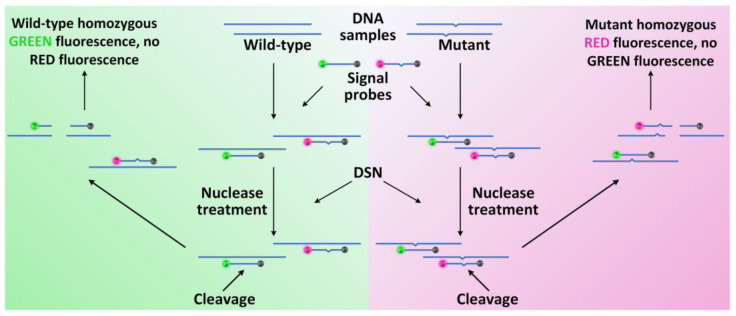
Scheme of the common DSNP assay for SNP detection. The green luminescent ball represents the first fluorescent donor, the red luminescent ball represents the second fluorescent donor, and the gray ball represents the fluorescent quencher. Reproduced with permission from Ref. [100].

DSN, which was first discovered in the Kamchatka crab, prefers to cleave DNA with double strands and DNA with double strands in DNA-RNA hybrid duplexes over single-stranded DNA. Additionally, this enzyme cleaves shorter, perfectly matched DNA duplexes at a rate that is noticeably higher than shorter, unmatched duplexes of the same length. “Duplex-specific nuclease preference” (DSNP) is a unique trait that enables the development of novel assays or the detection of SNPs in DNA. In this innovative assay, the PCR product is combined with DSN and signal probes after the DNA region containing the SNP site is amplified (FRET-labeled short-sequence-specific oligonucleotides). DSN only splits perfectly matched duplexes between the DNA template and signal probe during incubation to produce sequence-specific fluorescence. As shown in Figure 6, the employment of FRET-labeled signal probes coupled with the specificity of DSN presents a simple and efficient method for common SNPs’ detection [100].

### 3.1. DSN Platforms for SNP-Related Cancer Biomarkers’ Detection

The increasing evidence reveals that SNPs can influence cancer susceptibility and may affect the prognosis of patients with various cancers. Familial clustering and twin studies have suggested that genetic susceptibility significantly contributes to breast cancer development, including many known loci, whose genes have high-penetrance mutations (especially *BRCA1* and *BRCA2*), *ATM*, intermediate-risk alleles in genes such as *CHEK2* and *PALB2*, and common low-penetrance alleles [101]. Breast cancer susceptibility is determined by a large number of susceptibility sites. However, progress has been slow in identifying relevant susceptibility sites. Michailidou et al. [102] performed an analysis of 9 genome-wide association studies, including 10,052 breast cancer cases and 12,575 control women of European ancestry, selected 29,807 SNPs for further genome typing, and identified 41 associated with breast cancer. SNP loci are associated with an increased cancer risk, with which specific SNPs in BRCA1/2 mutation carriers are directly associated.

**Figure 7:**
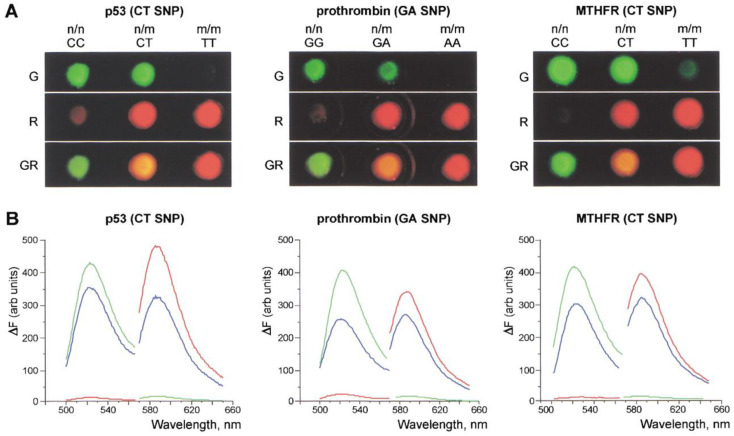
Investigation of *p53 C309T*, prothrombin *20210 G-to-A*, and *MTHFR C677T* polymorphous sites in homozygous and heterozygous DNA via the DSNP assay. (**A**) Images obtained with the fluorescent stereomicroscope equipped with green (G) and red (R) filters. GR: computer superposition of images obtained with green and red filters; n/n: homozygous DNA samples comprising wild-type sequence variant; n/m: heterozygous DNA samples; m/m: homozygous DNA samples comprising mutant sequence variant. (**B**) Normalized emission spectra of these samples obtained by the spectrofluorometer, with excitation wavelengths at 480/550 nm for green/red fluorescence, respectively. Green line: homozygous DNA samples comprising the wild-type sequence variant; red line: homozygous DNA samples comprising the mutant sequence; blue line: heterozygous DNA samples. Reprinted with permission from Ref. [100].

Altshuler et al. [103] proposed a DSNP method using DSN from the king crab. The method was used to study SNPs in the deletion of the *BRCA1* gene. It has been shown that DSNP is effective for counting the number of mutant alleles present in DNA samples. There was also a method published for finding several nearby SNPs in the *kRAS* gene at once. The strategy was successful in creating test methods for finding SNPs in additional human genes linked to cancer (*nRAS*, *hRAS*, and *p53*) (Figure 7).

### 3.2. DSN Platforms for SNP-Related Neurodegeneration Biomarkers’ Detection

Schizophrenia is a common nervous disease that often occurs among young and middle-aged people. Generally, there is no disorder of consciousness and intelligence, but it is manifested as a partial or complete loss of self-control, as well as a disorder of thinking and association, emotional, and volitional behavior disorder [104]. The *COMT* gene encodes catechol-O-methyltransferase, which is located on chromosome 22 at position 22q11.21. The full length of the *COMT* gene is 28,141 bp, and the full length of mRNA is 1289 nt, encoding a protein composed of 271 amino acid residues. COMT is the main enzyme in the metabolism of catecholamines as a ubiquitous enzyme in the body. It catalyzes the methylation of the third hydroxyl group of catecholamine in the presence of magnesium ions, which is considered to play an important role in the pathogenesis of schizophrenia [105]. The amino acid at position 158 of the COMT enzyme changed from valine (Val) to methionine (Met) and can induce the thermal instability of the enzyme, resulting in a 3-4-fold decrease of enzyme activity. The differences in *COMT* gene polymorphisms among individuals of different races are related to the genetic susceptibility to mental diseases and Parkinson’s disease [106].

Using the DSN detection technique, nine described human DNA samples expressing the rs16559 *COMT* gene SNP were analyzed. Two distinct primers were used in the experiment: one for the *COMT* gene’s wild-type sequence (COMT A1) and the other for its SNP-containing variant (COMT C) [107]. Both distinct primers contained a specific sequence that was the same as the fluorescent probe UZ-Fam and measured 16 or 15 nt in length, respectively. The DNA segments used in the analysis, which were amplified using COMT-out-dir and COMT-out-rev primers, contained the *COMT* gene’s rs16559 SNP either in homozygous or heterozygous form. The results showed that DSN detection could reliably distinguish between allelic variants in heterozygous and homozygous samples. The novel combination of DSN detection with allele-specific PCR was tested using the same model system. This modification involved two SPs, COMT-A2 and COMT-G, and a single forward primer, COMT-out-dir, to amplify fragments that contained the rs16559 SNP. This method demonstrated the flexibility of DSN detection for the quick identification of target fragments in a complicated PCR product in several model experiments. This technique can also be used to find SNPs using a standard short probe and is less expensive than other analysis methods.

Similar nervous disorder problems were brought on by the C677T polymorphism in the *MTHFR* (5,10-methylenetetrahydrofolate reductase) gene (Figure 7). The *ApoE* (apolipoprotein E) gene variations that were linked to the onset of Alzheimer’s disease were examined using DSNP, which either determined the predisposition to their origination or were implicated in the development of diseases [100,103].

### 3.3. DSN Platforms for SNP-Related Virus Detection

Persistent hepatitis B virus (HBV) infection can result in chronic hepatitis, cirrhosis, and primary liver cancer [108]. Taking anti-HBV medications is the most crucial and effective way to stop viral replication. To treat chronic hepatitis B (CHB), lamivudine and other anti-HBV drugs have been extensively utilized. However, drug resistance following prolonged therapy has emerged as a contentious issue in clinical therapy as a result of virus gene mutation. For instance, primary lamivudine resistance for HBV is linked to two point mutations in the polymerase gene’s tyrosine-methionine-aspartate-aspartate (YMDD) motif, that results in either rtM204V or rtM204I substitutions [109]. The numerous techniques that are currently being developed for the detection of particular gene mutations include bio-bar codes, branched DNA signal amplification testing/technology (NAT), allele-specific DNA hybridization, ligation or primer extension, electrochemical detection, and binary DNA probe assays [110,111]. However, only a few of these techniques are appropriate for clinical application.

Unmodified AuNPs and other materials were fabricated by Liu and colleagues to create a quick and accurate method for detecting single-base mismatches. Using two serum samples containing the HBV genomic DNA of patients, S1 nuclease/DSN was used to detect SNPs. They decided to find the point mutation rtM204V, which can result in drug resistance, to show biological validation of the method. The approach was based on nucleases’ high capacity for mismatch discrimination and nucleoside monophosphates’ superior stabilization of unmodified AuNPs over ssDNA and dsDNA [112].

**Figure 8:**
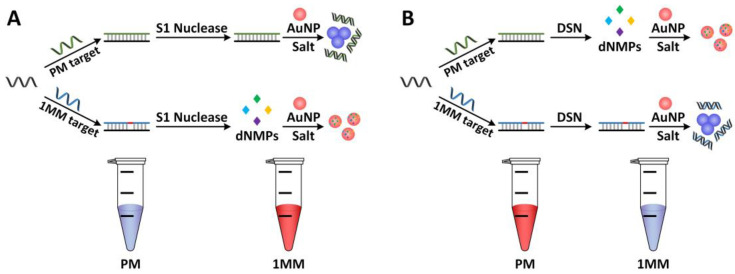
Schemes for detecting single-base mismatches without using optical labels. (**A**) The scheme was illustrated in the case of the S1 nuclease, and various DNA structures were used. S1 nuclease hydrolyzes mismatched DNA duplexes to 5′-phosphoryl-terminated products, but not perfectly matched duplexes. (**B**) Diagram of the DSN-based, label-free optical detection of SNPs. Lower left: images of the AuNP solution containing the probe, S1 nuclease, and various targets (PM or 1MM). Images of AuNP solution containing a probe, various targets, and duplex-specific nuclease are shown on the right bottom (DSN). The assays were incubated at 65 °C for 7.5 min before adding AuNP and the salt solution. Photographs were taken 2 min after a 40 µL salt solution was added. Reproduced with permission from Ref. [112]. Copyright 2011, Elsevier.

In contrast to S1, DSN prefers dsDNA over ssDNA by a factor of 1000 (Figure 8). This method revealed a striking color difference between the single-base mismatched target and the perfectly matched target 1MM (AC). The UV-vis spectra after 7.5 min of incubation at 65 °C showed a DF of 5.2. Since the assays had a DF of 9.1 after 15 min of incubation, a time scale was also used to enhance discrimination. Theoretically, the DSN-based method is effective for longer ssDNA targets because DSN only selectively cleaves the dsDNA region, and the non-hybridized region will not prevent the detection of the interested sequences. The ability to detect a single mismatch at any position on the targets with a length of up to 80 bases has been demonstrated, and this could make it easier to design the primers needed for the necessary amplification step for genomic samples. The technique for identifying single-base mismatches in actual genetic samples showed promise for clinical applications (HBV genomic DNA samples).

## 4. Conclusions and Outlook

Owing to the close associations between miRNA and SNP and various diseases, there is an urgent need to construct prompt, highly sensitive, and specific arrays. Due to the unique enzymatic activity of DSN, the current breakthroughs in miRNA and SNP detection based on DSN-coupled signal amplification approaches have been critically and comprehensively reviewed here. Significant efforts have been made to improve miRNA detection, and a range of improved or new techniques have been discussed. We have provided insights into the functioning mechanism, analytical range, and multiplex analysis of these sensing approaches for prominent biomarker detection in this study.

DSN is effective for miRNA and SNP detection, but there are some minor drawbacks in clinical applications. A hybrid substrate with at least 10 bp is necessary for the efficient cleavage of DSN. Due to its requirement for divalent cations, DSN is incompatible with some applications. The nonspecific cleavage of dsDNA hybrids makes multiplex detection incredibly difficult. It is important to note that DSN can only mediate linear signal amplification. Additional techniques are thus needed to improve analytical sensitivity for the detection of low-abundance miRNAs. Future DSN applications will concentrate on addressing and extending such important issues as advancements in chemical or material science.

Considering that DSN is duplex-specific rather than sequence-specific, multiplex detection of a panel of miRNAs requires additional specialized probes. The development of novel chemical modification and nanoparticle-based fluorescence labeling techniques will facilitate high-throughput miRNA detection. The quantity and density of the immobilized probes play a significant role in determining the sensitivity of DSN-mediated signal amplification, in contrast to conventional assays that depend on optimized coating probe concentrations. Overcrowding of the probes reduces the detection signal due to steric hindrance between the probes. DSN assays hardly ever encounter steric hindrance because the trace target miRNA preferentially binds the peripheral probes up until all the probes are cleaved. Investigating new coating techniques that enable the conjugation of more probes on the chips would, in this case, significantly enhance the detection performance. Nanomaterials that could be used to increase the detection signal’s strength or the number of probes include QDs, graphene, carbon nanotubes, and gold nanoparticles. Due to its capacity for the highly sensitive and specific detection of miRNA and SNP biomarkers, DSN is a promising biomedical tool for elucidating the physiological mechanisms that regulate disease formation and for facilitating disease diagnosis and prognosis.

## Data Availability

Not applicable.
